# Imaging Review of Skeletal Tumors of the Pelvis Malignant Tumors and Tumor Mimics

**DOI:** 10.1100/2012/240281

**Published:** 2012-04-19

**Authors:** Gandikota Girish, Karen Finlay, David Fessell, Deepa Pai, Qian Dong, David Jamadar

**Affiliations:** ^1^Department of Radiology, University of MI, 1500 E. Medical Center Drive, TC-2910, Ann Arbor, MI 48109-0326, USA; ^2^Department of Radiology, Henderson General Hospital, Hamilton Health Sciences and McMaster University, 711 Concession Street E, Hamilton, ON, Canada L8V 1C3

## Abstract

Malignant lesions of the pelvis are not uncommon and need to be differentiated from benign lesions and tumor mimics. Appearances are sometimes nonspecific leading to consideration of a broad differential diagnosis. Clinical history, anatomic location, and imaging characterization can help narrow the differential diagnosis. The focus of this paper is to demonstrate the imaging features and the role of plain films, computed tomography, and magnetic resonance imaging for detecting and characterizing malignant osseous pelvic lesions and their common mimics.

## 1. Introduction

 Hemopoietic marrow predominates in the bony pelvis until late in life. Metastatic disease, plasma cell myeloma, Ewing's sarcoma, and lymphoma are among the tumors that primarily localize to hematopoietic marrow. The tendency for these neoplasms to involve both the axial and appendicular skeleton in the young and predominantly the axial skeleton in the middle aged and elderly is consistent with the changing distribution of red marrow that occurs with advancing age. The variability in the appearances of both primary and secondary pelvic tumors may lead to diagnostic difficulty, especially in the setting of differentiating primary tumors from metastasis. In the first part of this paper we discussed imaging techniques, the role of biopsy, and the influence of age in the differential diagnosis of tumors of pelvis. We also reviewed the radiological appearances of common benign bone tumors of the pelvis. The aim of this paper is to review the multimodality appearances of common malignant tumors of the bony pelvis and frequent mimics. 

## 2. Recent Advances

 Despite significant advances in other modalities, radiographs remain the mainstay for initial assessment and diagnosis of bone tumors [1, 2]. Current multidetector-row computed tomography (MDCT) is better than magnetic resonance imaging (MRI) in the assessment of bone tumor characteristics like subtle cortical breach and periosteal reaction and in classifying matrix mineralization [1]. MRI remains superior to CT in assessing the involvement of soft tissue, marrow, and neurovascular structures [2]. Limb salvage surgical techniques are now preferred to amputation because of equal or better long-term survival rates, feasibility of resection of distant metastasis, and improved functional results [2, 3]. Because of consideration of limb-sparing surgery, the biopsy tract (which needs to be resected along with the tumor as it may be contaminated) should not traverse the uninvolved compartments. Specifically, the gluteal musculature and rectus femoris should not be in the biopsy tract as these muscles need to be preserved for postoperative rehabilitation and stabilization of the prosthesis. It is ideal to discuss the biopsy approach with the referring oncological surgeon. A higher diagnostic yield can be achieved by targeting nonsclerotic, nonnecrotic areas and obtaining more tissue with larger bore needles and multiple passes [4, 5].

## 3. Malignant Tumors (below the Age of 40 Years)

### 3.1. Osteogenic Sarcoma (OGS)

Conventional OGS is second in frequency to multiple myeloma, as a primary malignant tumor of bone [6]. It occurs commonly in the second and third decades of life, with a second peak described in the literature usually attributed to malignant degeneration of Paget's disease or prior radiation. The pelvis is a relatively infrequent site for OGS, while the metaphysis of tubular bones of the appendicular skeleton is the most common site. 

**Figure 1:**
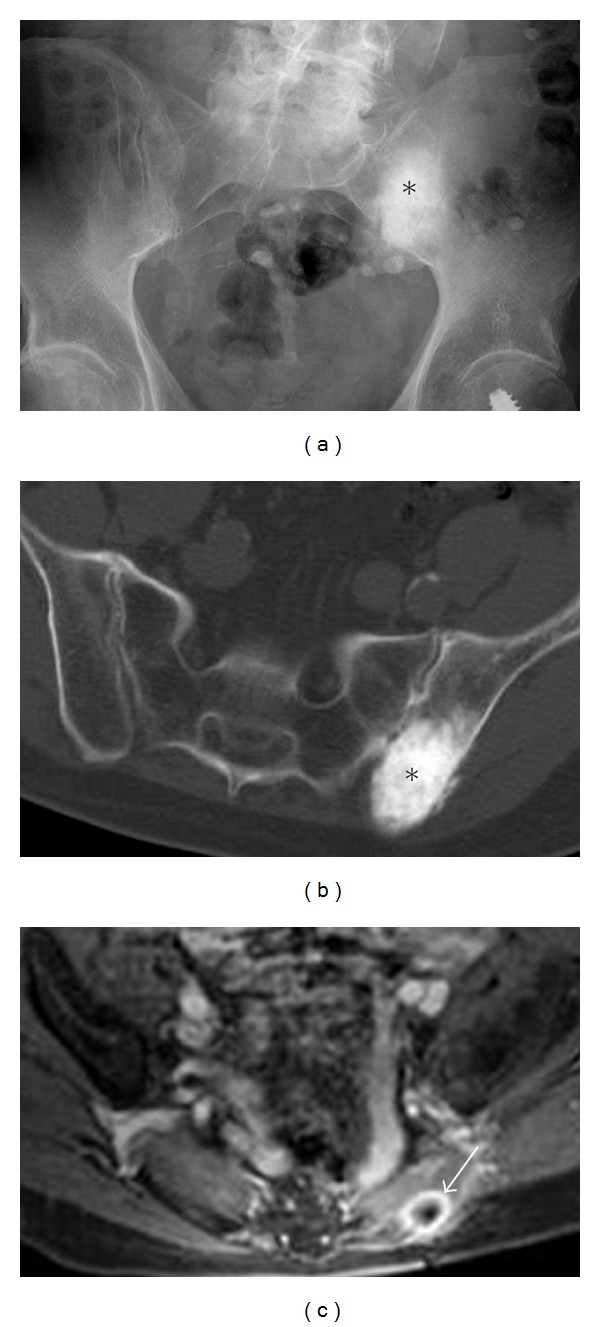
Conventional osteosarcoma. Elderly male presenting with a history of lower back pain diagnosed as conventional osteogenic sarcoma following biopsy. Anteroposterior (AP) radiograph (a) and axial CT scan (b) of the pelvis reveal aggressive bone-forming neoplasm involving the left ilium (asterisk). Follow-up axial T2 fat-saturated image (c) after tumor resection demonstrates an osseous metastasis with a characteristic peripheral halo (Arrow), which should not be confused with a metallic susceptibility artifact.

**Figure 2:**
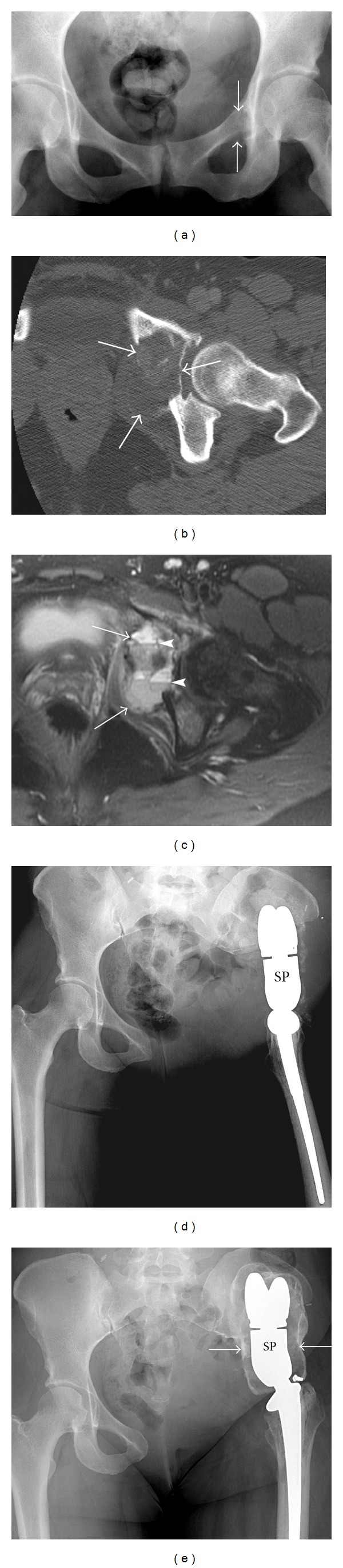
Osteolytic osteosarcoma. A 35-year-old female presenting with left hip pain diagnosed with aggressive lytic osteosarcoma. AP radiograph (a) demonstrates a subtle lytic lesion (arrows) in the left superior pubic ramus extending to involve the medial acetabulum. The extent of this predominantly lytic lesion with pockets of dense osteoid matrix is better appreciated on axial CT (arrows) (b). Axial T2 fat-saturated image (c) of the same lesion (arrows) depicts multiple fluid-fluid levels (arrowheads). The patient underwent left hemipelvectomy with placement of saddle prosthesis (SP) (d). A shell of heterotrophic ossification formed around the prosthesis (arrows) (e) is an expected finding, which helps stabilize the prosthesis and should not be misinterpreted as recurrence.

Characteristically, the tumor produces osteoid or immature bone. Radiographically there is extensive bone formation, often described as having a “cloudy” appearance (Figures 1(a) and 1(b)). Mixed areas of osteolysis and sclerosis are present. Predominantly osteolytic osteosarcomas are often a more aggressive type (Figures 2(a), 2(b), and 2(c)), where the abundant osteoid matrix has not mineralized. In the pelvis, a cortical breach and associated soft tissue masses are often present (Figure 2(b)) [7], and involvement of flat bones may be more frequent in older patients. Plain radiographic appearances remain the gold standard in the specific diagnosis of OGS. The role of MR is to define the extent of the neoplasm, associated soft tissue masses and relationship to adjacent neurovascular structures, with a view to planning management. Care must be taken not to obscure the tumor/marrow interface by using T1 post-gadolinium sequences without fat suppression. MR typically reveals a large soft tissue component, with heterogeneous high and low signal on both T1 and T2 [7, 8], and fluid-fluid levels can be appreciated in osteosarcomas (Figure 2(c)). Secondary aneurysmal bone cyst formation is encountered in a wide variety of benign and malignant bone neoplasms in addition to widely known giant cell tumor and telangictatic osteosarcoma [9, 10]. It has been noted that the most common malignant tumor demonstrating fluid-fluid levels is the conventional osteosarcoma and the most common benign lesion is an aneurysmal bone cyst [9, 10]. Preoperative chemotherapy has dramatically improved the survival rates of osteogenic sarcoma.

### 3.2. Ewing's Sarcoma (ES)

**Figure 3:**
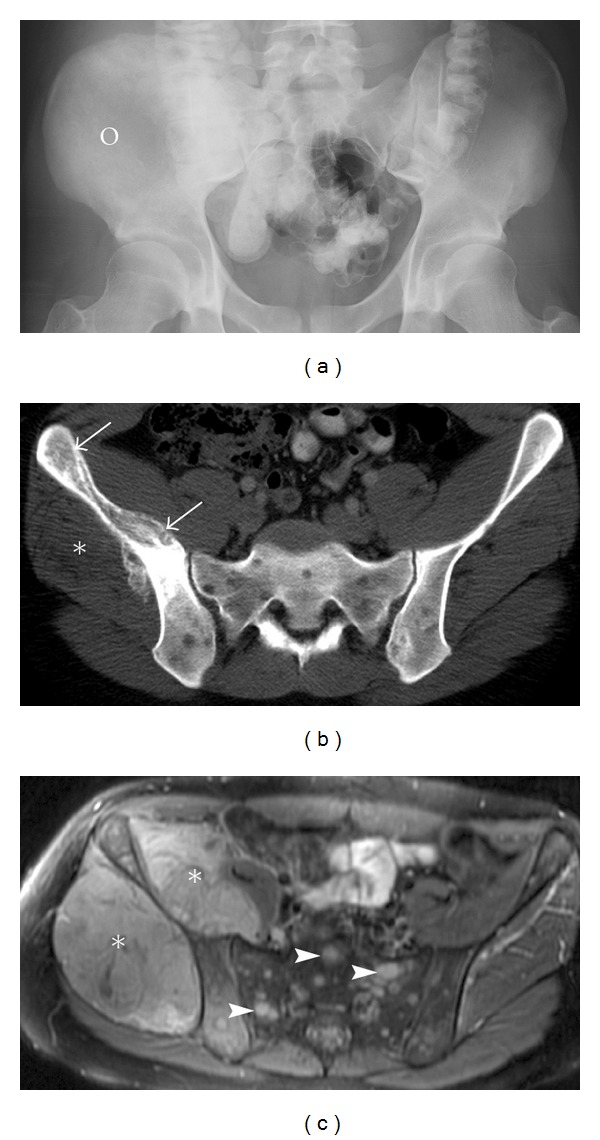
Ewing's sarcoma. A 21-year-old male with advanced Ewing's sarcoma. AP radiograph (a) of the pelvis reveals a vague mixed density abnormality diffusely involving the right ilium (circle). Note the asymmetry when compared to the normal left ilium. Incidental note is made of oral contrast in the bowel. CT scan (b) of the pelvis demonstrates permeative lesion of the right ilium (arrows) and an associated soft tissue mass (asterisk). Small multifocal lytic lesions in the sacrum and bilateral ilium are in keeping with metastasis. Axial T2 image (c) of the pelvis reveals a large exophytic soft tissue mass (asterisk) along the anterior and posterior borders of ilium. Note the predominance of a huge soft tissue mass when compared to the bone abnormality, characteristic of small round blue cell tumors. Marrow edema is noted in the affected right ilium and multiple T2 bright lesions (arrowheads) in the sacrum and right ilium represent metastasis.

The pelvis or sacrum is involved in 20% of ES cases. 75% of patients are between 10 and 25 years old [8], while, in those under 5, metastatic neuroblastoma is more probable. They often present with subtle plain film findings (Figure 3(a)), where ill-defined, permeative, or “moth-eaten” bone destruction is a major feature of ES best appreciated on CT (Figure 3(b)) [8]. Periostitis is often exuberant, frequently displaying an onionskin or laminated appearance. The soft tissue element may be extremely large, especially in pelvic disease (Figures 3(b) and 3(c)). The mass may contain calcifications and lead to diagnostic confusion, raising the possibility of osteosarcoma or chondrosarcoma. Due to the wide spectrum of radiographic appearances, ES can mimic most malignant and a few benign entities, including osteomyelitis, eosinophilic granuloma, and giant cell tumor [8]. A useful discriminator is the presence of benign periostitis, which should lead one to consider the benign entities listed above. ES is often associated with a characteristic large soft tissue component, often serving as a clue to the correct diagnosis.

## 4. Malignant Tumors (above the Age of 40 Years)

### 4.1. Metastases

Metastases are the most common malignant neoplasm of the pelvis, specifically the sacrum, with lung, breast, kidney, and prostate carcinoma the most frequent primary neoplasms [11]. Metastatic lesions are usually osteolytic although sclerotic lesions can be encountered from primary tumors, such as prostate or breast carcinoma. 

**Figure 4:**
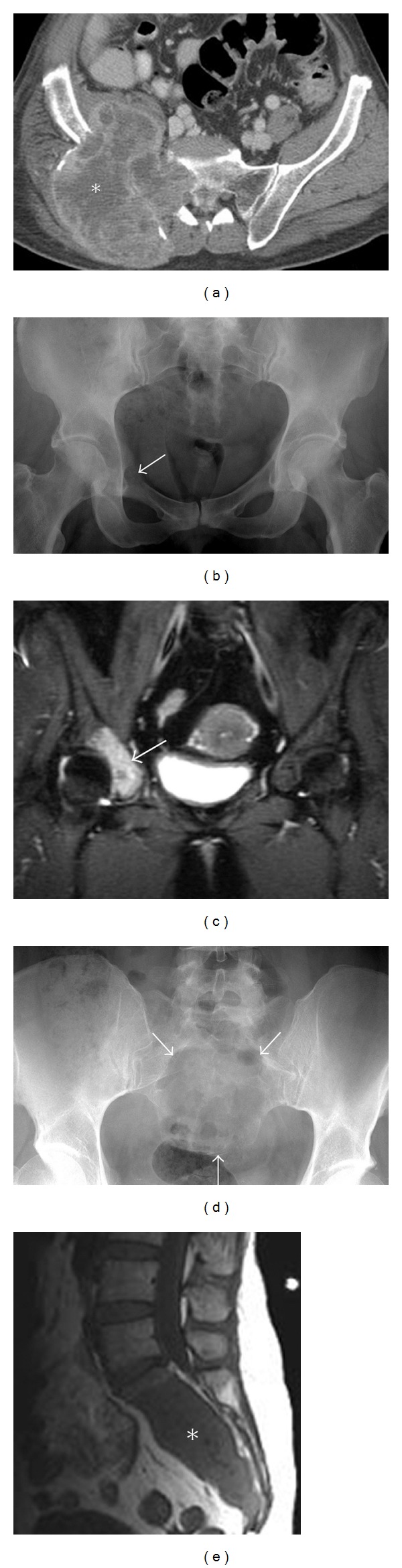
Metastasis. A 58-year-old male presents with a short history of right hip pain and a remote history of primary renal cell carcinoma diagnosed with metastases. Axial post-contrast CT of the pelvis (a) demonstrates a large destructive expansile necrotic mass (asterisk), arising within the right ilium and extending into the right sacrum. Two different patients with breast cancer metastasis to the acetabulum (b) and (c) and to the sacrum (d) and (e). These lytic lesions can be difficult to identify on plain films (b) and (d) more easily appreciated on coronal STIR (c) and sagittal T1 (e) MRI. Note the destruction of arcuate lines by the sacral lesion; destruction of the subarticular cortex, asymmetric density of the right acetabulum, and fuzziness of the right iliopectineal line by the acetabular metastasis.

Skeletal metastases of primary renal origin are common, as 25–30% of patients with renal carcinoma develop skeletal metastases [12]. Infrequently, this occurs in the absence of metastases to other organ systems, and renal cell carcinoma must be strongly considered in the differential diagnosis of any skeletal metastatic tumor of unknown primary. These patients are usually over 40 years old, with the average age of 55 years [13]. Renal cell carcinoma has several unique features that increase its significance for early recognition. First, the metastatic deposits may occur many years after the primary tumor has been treated. As such, patients who are thought to have received curative resection need to be monitored for up to ten years for possible bone metastasis. Second, the pelvis is a typical metastatic site, and, given that lesions are often solitary, an en bloc surgical resection may offer the opportunity for a curative surgical treatment in select patients. Renal cell metastases appear extremely aggressive on imaging, typically demonstrating osteolysis and in some cases are extremely expansile, presenting as characteristic “blow-out” lesions (Figure 4(a)). Septations and a soft tissue mass are additional features, and lesions are typically hypervascular and have a tendency to bleed following biopsy [14, 15]. MDCT is more sensitive and specific in detecting renal metastasis than FDG PET or bone scan [16]. 

Lytic pelvic metastasis can be difficult to identify on plain films (Figures 4(b) and 4(d)) but are more easily appreciated on MRI (Figures 4(c) and 4(e)) due to obvious replacement of marrow fat by fluid-sensitive tumor tissue. Appreciating asymmetry between the two halves of the bony pelvis on plain films helps in identifying subtle lesions. Bone scans are very sensitive in the detection of osteoblastic metastasis, less accurate in detecting lytic metastasis, and not helpful in diagnosing multiple myeloma. A bone scan can be misinterpreted when performed for assessment of tumor response following treatment because of the flare phenomenon which shows a paradoxical increased uptake in a healing metastasis, up to 6 months following treatment [17, 18]. In many tumors, FDG-PET is more sensitive than a bone scan in diagnosing metastasis and assessing tumor response after therapy; however, the availability and cost of FDG-PET are also limiting factors. Despite lower sensitivity, bone scans are still valuable in detecting thyroid bone metastases and cannot be completely replaced by FDG PET [19].

### 4.2. Plasmacytoma/Multiple Myeloma

**Figure 5:**
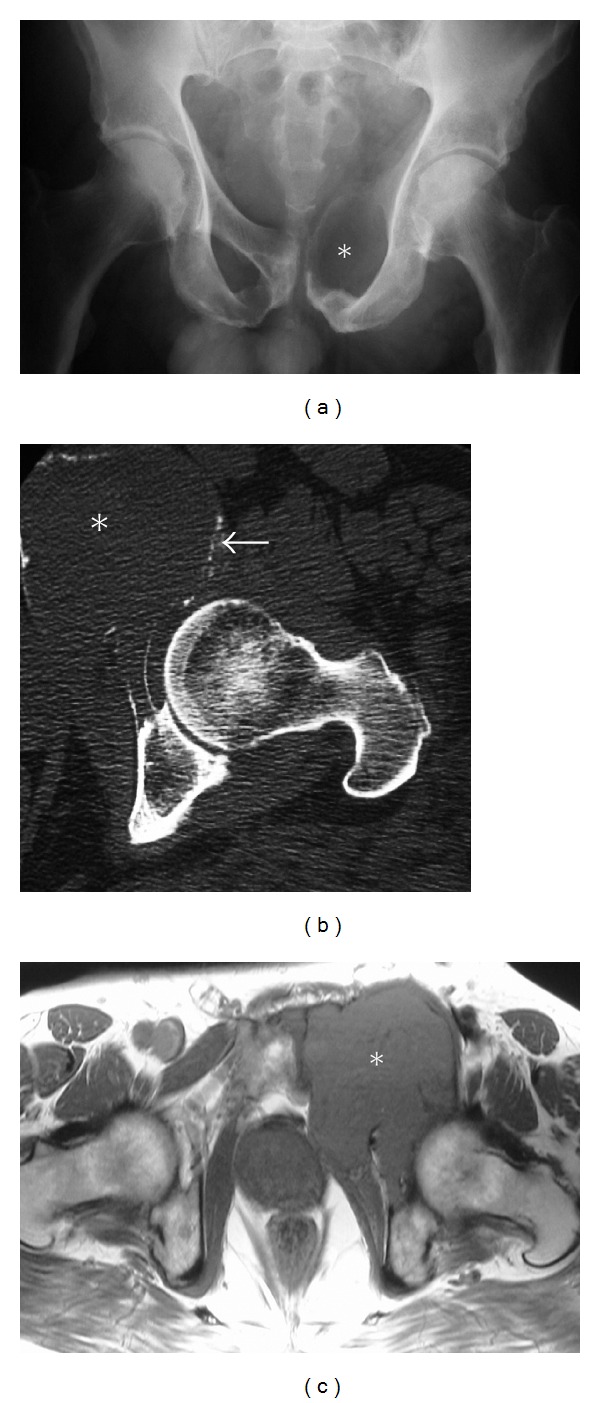
Plasmacytoma. A 68-year-old male with plasmacytoma. Radiographs (a) of the pelvis showing expansion of the left obturator foramen and an expansile osteolytic lesion involving the superior pubic ramus (asterisk). Axial CT (b) depicts the large lytic mass expanding the superior pubic rami (asterisk). A thin mineralized periphery in keeping with expanded cortex is appreciated on CT (arrow). Axial T1 MR (c) demonstrates the expansile mass (asterisk) displacing the left femoral vessels, indenting the left anterolateral aspect of the prostate and invading the adjacent obturator internus muscle.

Multiple myeloma (malignancy of monoclonal B cells) is the most common primary bone neoplasm in the elderly. Solitary plasmacytoma is rare, compared to multiple myeloma, and comprises less than 5% of plasma cell dyscrasias. The affected age group is about 50 years, younger than for multiple myeloma [20]. Similar to myeloma, the pelvis is a common site of disease, after the axial skeleton [21], and the radiographic appearances are variable. A plasmacytoma is commonly considered a mimic of other lytic bony lesions and may appear as a multicystic expansile lesion (Figure 5), with thickened trabeculae, or a purely osteolytic focus without expansion. This latter appearance may indicate amyloid deposition. If expansile, these lesions can also simulate a giant cell tumor. Rarely, calcification or even ossification can occur within a plasmacytoma, mimicking osteosarcoma or chondrosarcoma [7, 21]. Distinction from the expansile metastases seen in renal and thyroid malignancy can be equally difficult.

**Figure 6:**
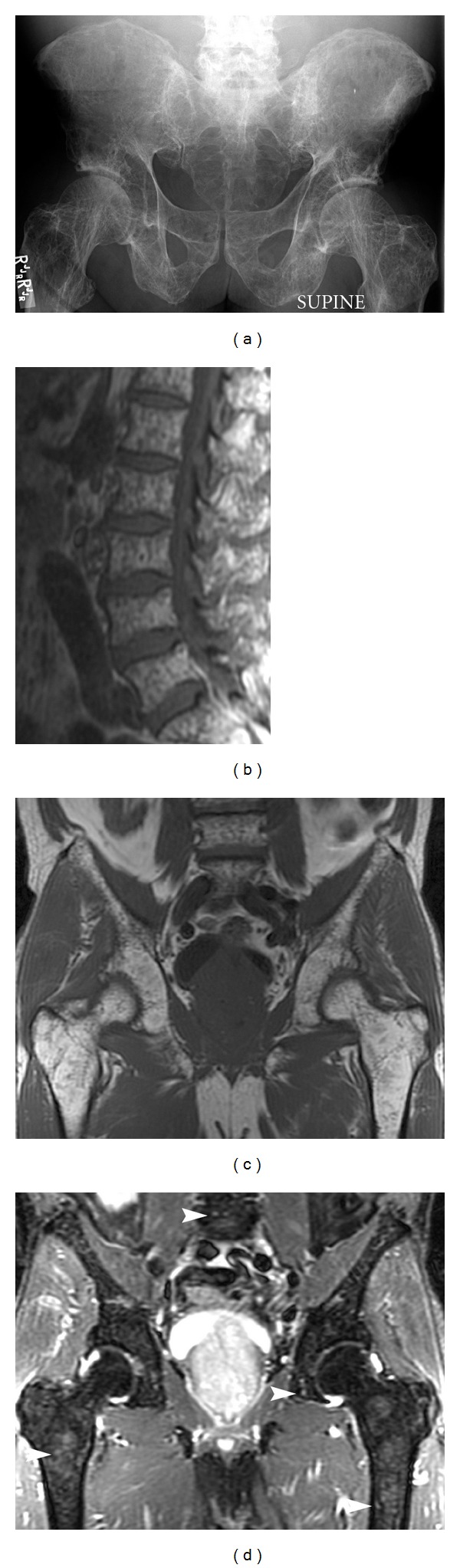
Multiple myeloma. Elderly patient with multiple myeloma. Plain film (a) demonstrates extensive osteopenia and multiple punched-out lytic lesions involving the entire pelvis. Sagittal T1 (b) of the lumbar spine, coronal T1 (c), and coronal STIR (d) demonstrate innumerable T1 hypointense and T2 hyperintense lesions (arrowheads) in the medullary cavity of the entire visualized skeleton.

Osteopenia and punched-out lytic lesions are hallmarks of multiple myeloma on the plain film (Figure 6(a)). On MRI, multiple myeloma can present as distinct multifocal small intramedullary lesions, diffuse marrow abnormality, a variegated appearance of the marrow, or a combination of the above (Figures 6(b), 6(c), and 6(d)). MRI is helpful in staging the disease and identifying any complications. Whole-body MRI can stage the disease more accurately as it has the potential to demonstrate up to 50% more lesions when compared to traditional spinal imaging alone [22].

### 4.3. Lymphoma

Primary lymphoma of bone is uncommon, comprising approximately 3% of primary bone tumors [23]. The majority of these are non-Hodgkin's lymphoma, with Hodgkin's variety occurring in less than 5% of cases [24].

**Figure 7:**
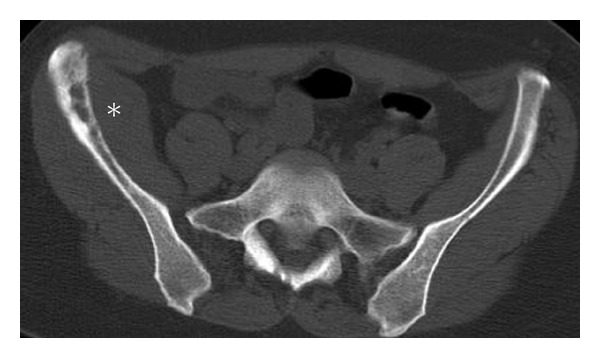
Lymphoma. A 30-year-old male with lymphoma. Axial CT scan illustrating permeative lesion of the right ilium with associated large soft tissue mass (asterisk) and relatively intact intervening cortex, a hallmark of small round blue cell tumors like lymphoma.

Imaging characteristics of lymphoma can be fairly nonspecific and hence are considered in the differential diagnosis for a wide variety of neoplasms. Imaging features include permeation of the cortical bone, often without frank cortical destruction and an associated soft tissue mass (Figure 7). Spread along the long axis of the bone, involvement of the marrow without frank overlying cortical destruction is not uncommon. Lymphoma can show transarticular spread, a finding not commonly seen in other lesions. The majority of these tumors are low in signal intensity on both T1-and T2-weighted sequences; however, it is now accepted that they may have a variable appearance on T2-weighted imaging [25].

### 4.4. Chondrosarcoma

**Figure 8:**
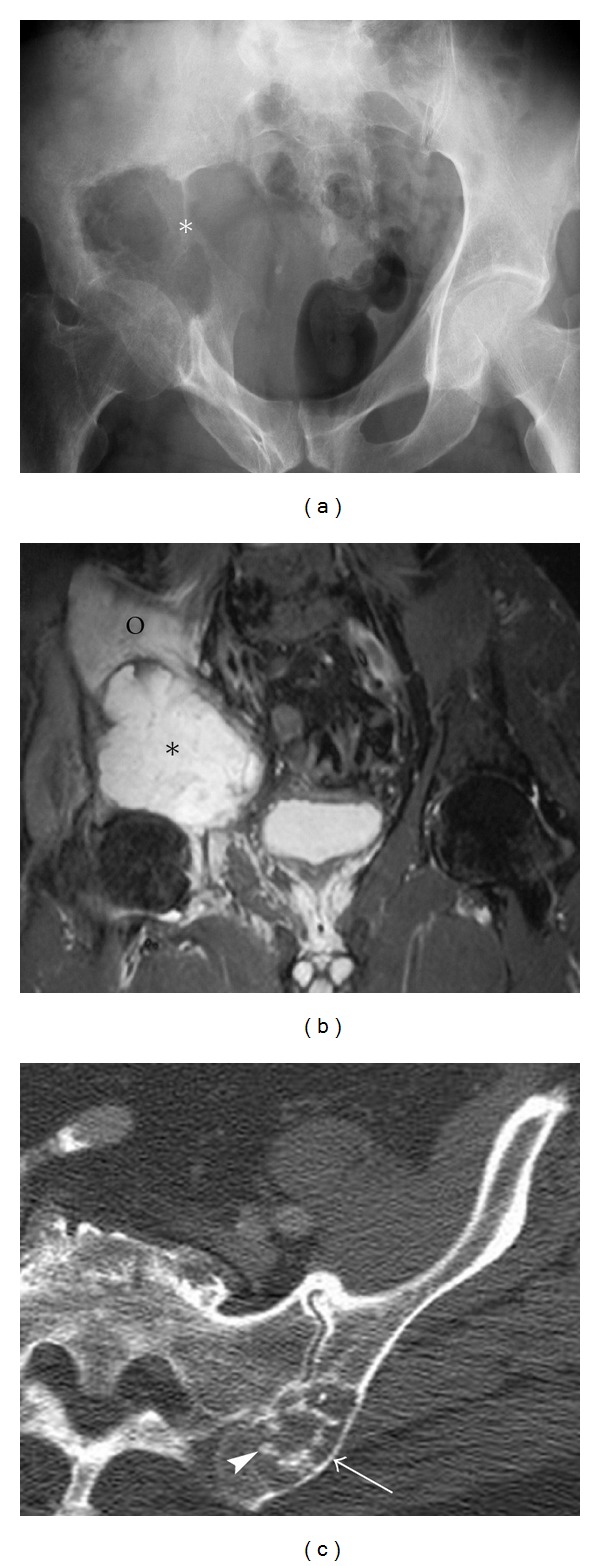
Chondrosarcoma. A 62-year-old male presents with right hip pain, diagnosed with chondrosarcoma. Pelvic radiograph (a) outlines a lytic destructive abnormality centered on the superior acetabular region (asterisk). An associated soft tissue mass is seen extending into the pelvis. The relative lack of calcification of the chondroid matrix would be in keeping with a high-grade chondrosarcoma. Coronal STIR (b) demonstrates the destructive mass (asterisk) as well as associated marrow edema within the right iliac bone (circle) and a small right hip joint effusion. Axial CT scan of another patient demonstrates more typical appearances of chondrosarcoma with endosteal scalloping (arrow) and chondroid matrix (arrowhead) (c).

Chondrosarcoma usually presents in individuals over the age of 40 years [7] and rarely occurs in children. Occasionally this entity is diagnosed after malignant degeneration of an osteochondroma, usually in cases of multiple osteochondromatosis. Consequently, rapid growth of an osteochondroma and/or its cartilage cap is an ominous sign, requiring surgical resection of the lesion. Pain, with or without a soft tissue mass, is the most common clinical presentation. Imaging features cannot always reliably differentiate an enchondroma from a low-grade chondrosarcoma, but fortunately low-grade chondrosarcomas grow very slowly and rarely if ever metastasize. On clinical presentation, pain occurs more commonly in chondrosarcoma, whereas enchondromas are usually asymptomatic. MRI findings that suggest chondrosarcoma include associated soft tissue mass or soft tissue edema, cortical destruction, periosteal reaction, adjacent marrow edema, lack of intervening marrow fat within the lesion, and endosteal scalloping involving more than two-thirds of the peripheral cortex thickness [26, 27]. An enchondroma may also show endosteal scalloping but generally this involves less than two-thirds of the cortical thickness. As expected in a chondrosarcoma, an ill-defined border and wide zone of transition is indicative of high-grade histology often associated with large areas of myxoid, noncalcified matrix (Figures 8(a) and 8(b)) [7, 26]. The size of the associated soft tissue mass is a less useful predictor of malignant potential. Tumoral calcification is a hallmark feature, but patterns of calcification are variable. Chondroid matrix and endosteal scalloping are better appreciated on CT (Figure 8(c)), and the density of calcification relates to the degree of malignancy: well-organized calcification usually signifies a low-grade tumor, whereas high-grade tumors typically demonstrate amorphous, irregular, and scattered calcifications.

### 4.5. Chordoma

**Figure 9:**
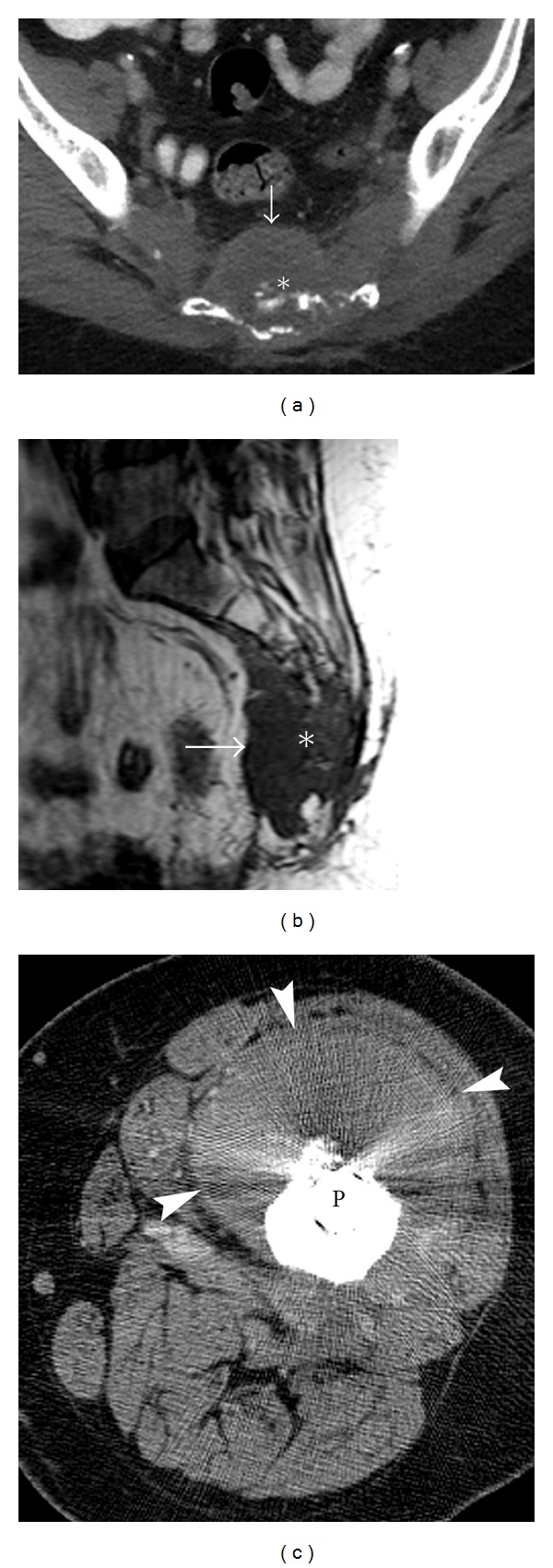
Chordoma. A 64-year-old female presenting with low back pain, diagnosed with an advanced sacral chordoma. Axial CT (a) and sagittal T1 midline image (b) demonstrate a large midline mass with internal calcification, destroying the sacrum (asterisk) and infiltrating the presacral space anteriorly and epidural space posteriorly. Axial CT (c) showing a large soft tissue metastasis (arrowheads) in the thigh around the femur. Hardware artifact is from an intramedullary rod (P) internally fixing the pathological fracture of the femur.

Chordoma is the most common primary malignant sacral neoplasm, accounting for approximately 20–34% of sacral lesions [7]; however, chordomas are relatively rare, accounting for only 2–4% of all primary malignant bone tumors. These neoplasms occur in older adults, with a mean age of 50 years [7, 21], and the ratio of male to female is two to one. Arising from notochord remnants, chordomas have very characteristic locations. One-half of cases occur in the sacrococcygeal region and one-third occur at the base of the skull. Transverse processes of vertebrae and the paranasal sinuses are less common sites [28]. Because chordomas typically exhibit slow growth, they are often large masses at time of diagnosis. On plain films, a chordoma appears as a solitary midline lesion with bony destruction. A soft tissue mass is always present, and approximately 50% of these contain focal calcifications (Figure 9(a)) [29]. CT and MRI help demonstrate the soft tissue component, calcifications, and epidural extension. On MRI, typical chordomas are iso- to slightly hypointense to skeletal muscle on T1-weighted images and usually hyperintense on T2-weighted images (Figure 9(b)) [21, 30], thought to be due to the intratumoral accumulation of mucin. Radical resection is the treatment of choice but they frequently recur and can metastasize (Figure 9(c)).

### 4.6. Postradiation Sarcoma

**Figure 10:**
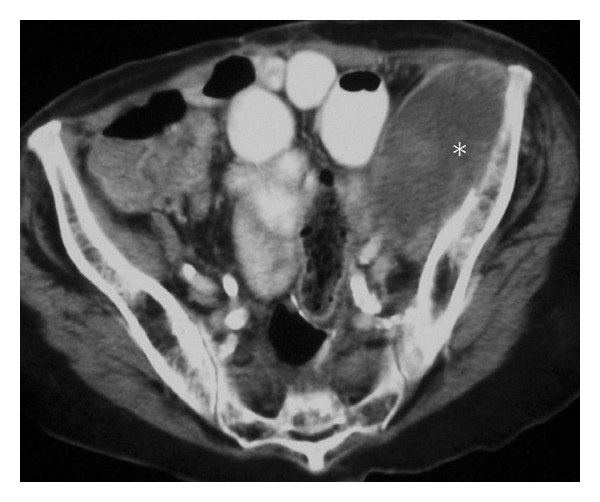
Post-radiation sarcoma. A 78-year-old female presenting with left upper thigh and hip pain. The patient had received radiotherapy to the pelvis 32 years previously, for an ovarian malignancy, and was later diagnosed with radiation-induced sarcoma. Axial contrast-enhanced CT identifies a large, heterogeneously enhancing mass along the inner surface of the left ilium (asterisk) with local bone destruction of the inner cortex.

Radiation-induced neoplasms may form in the areas that receive a sufficient dose to induce mutation, but not enough to destroy the regenerative capacity of the bone or soft tissue. The average latency period is 11 years, and the overall incidence is thought to be quite small, not exceeding 0.5% [31]. Chemotherapy when combined with radiation is associated with increased risk of postradiation sarcoma. The criteria for diagnosis of post-radiation sarcoma include tumor arising within the radiation field, a long latent period, histological proof, and a different histological pattern from the primary tumor [31]. A three- to four- year latency period is generally suggested as a minimum cutoff to ensure histological independence. Postradiation sarcomas most commonly arise from bone within the radiation field applied for the treatment of adjacent soft tissue pathology. Osteosarcoma is the most common postradiation sarcoma (Figure 10), and the most common soft tissue postradiation sarcoma is malignant fibrous histiocytoma. Chondrosarcoma can also occur [31]. Finally, radiotherapy has also been implicated in various fractures of the pelvis and sacrum.

## 5. Conditions Which May Mimic Neoplastic Process

### 5.1. Hemophilic Pseudo Tumor

**Figure 11:**
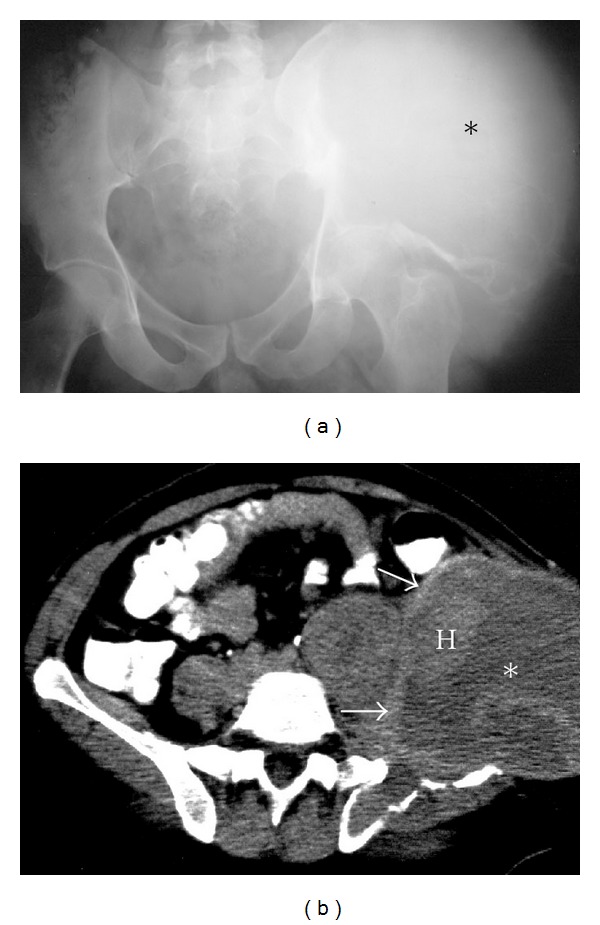
Hemophilic pseudo tumor. A 60-year-old male with hemophilic pseudo tumor of the left iliac wing. Plain radiograph (a) of the pelvis illustrating a large mass lesion (asterisk) destroying the left ilium and the acetabulum. Axial CT (b) of the pelvis demonstrates a large soft tissue density mass, centered within the left ilium (asterisk). Several areas of heterogeneous density (H) representing recent bleeds are present within the mass. Close inspection reveals a thin ballooned cortex (arrows) of the affected ilium.

Initial impressions of this entity can be misleading. Misdiagnosis of a hemophilic pseudo tumor as Ewing's sarcoma, skeletal metastases, or infection is well recognized. Earlier stages of subperiosteal hematoma can produce a periostitis simulating an aggressive process. An established intraosseous hematoma produces a lytic abnormality that may be mistaken for primary or secondary tumor-like lesions (Figure 11(a)). Characteristic radiographic findings include a large soft tissue mass with adjacent bone destruction (Figure 11(b)) [32]. Calcifications are also common, and often new bone formation at the edge of the lesion can result in periosteal elevation. In most cases accurate diagnosis is achieved with knowledge of the underlying disease.

### 5.2. Infection: Osteomyelitis

**Figure 12:**
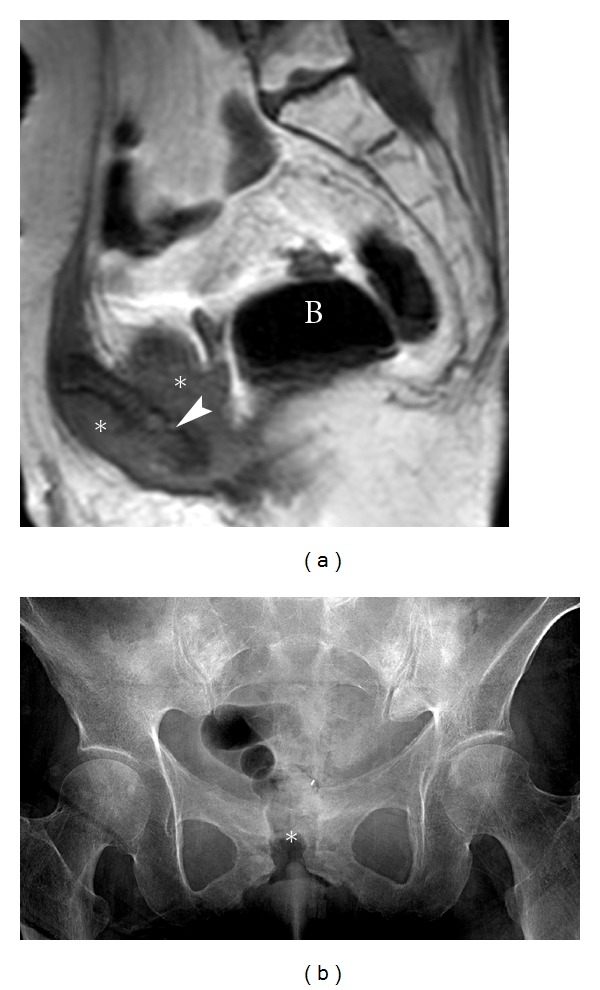
Pubic osteomyelitis. A 70-year-old female, with a history of previous surgery and radiotherapy for carcinoma of the cervix, presented with an open wound over the symphysis pubis. The clinical concern was the possibility of local tumor recurrence in the pubic symphysis region. The patient was subsequently diagnosed as having osteomyelitis. Sagittal T1 MRI (a) shows a soft tissue mass (asterisk) with underlying destruction of the symphysis pubis (arrowhead). Note the presence of air in the bladder (B) from a vesicovaginal fistula. Biopsy and culture proved the changes were due to osteomyelitis, rather than tumor recurrence. AP Radiograph (b) showing cortical irregularity and destruction in the region of pubic symphysis with widening of the joint (asterisk). Gas is projected within the symphysis pubis and adjacent soft tissues in keeping with the known vesicovaginal fistula.

Aggressive radiological features of osteomyelitis can mimic a neoplastic process, specifically when it presents with bone destruction and an associated soft tissue mass (Figure 12). As always, the clinical history is crucial. In addition, there is no predilection for location or age. When a bony sequestrum or gas is demonstrated, the differential diagnosis is significantly narrowed and osteomyelitis should be strongly considered [33].

### 5.3. Insufficiency Fracture

**Figure 13:**
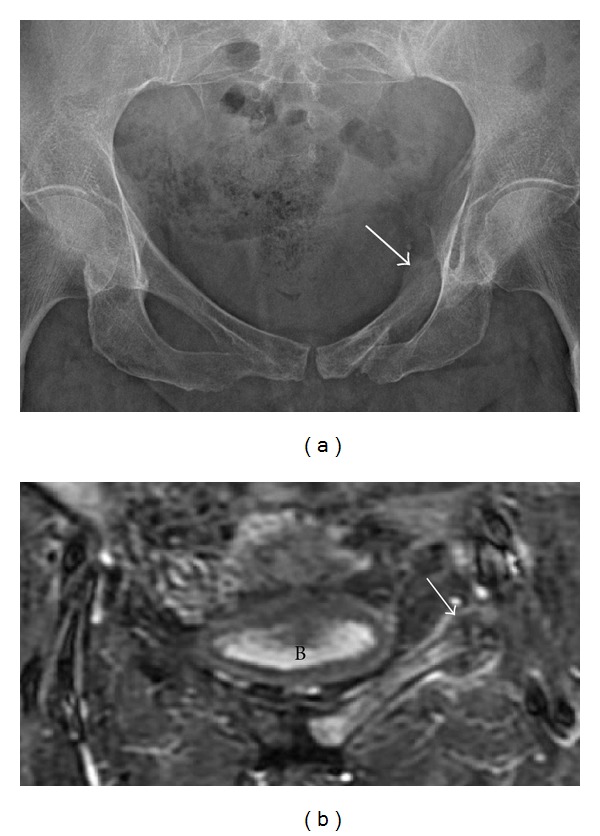
Insufficiency fracture. A 40-year-old male with a pelvic insufficiency fracture. Radiograph (a) demonstrates an expansile osseous lesion with periosteal reaction and maturing callus along the left superior pubic ramus (arrow). No definite fracture line is identified. Coronal STIR (b) of the pelvis demonstrates bone marrow edema throughout the left superior pubic ramus, fracture site, and surrounding callus (arrow). B: bladder.

Insufficiency fractures of the sacrum are a relatively common cause of lower back pain in elderly patients, particularly osteopenic women [34–38]. Insufficiency fractures may also occur following irradiation of the pelvis [39], rheumatoid arthritis, Paget's disease, osteomalacia and renal osteodystrophy. Signs and symptoms are often nonspecific, and the diagnosis is usually not initially considered. Findings on plain films can be subtle and include ill-defined vertical oriented lines unilaterally or bilaterally through the sacral ala and disruption of the sacral arcs. Bone scan may reveal typical bilateral sacral uptake in the classic H-shape, which assists in making the correct diagnosis. The healing process of insufficiency fractures involving the pubic rami can lead to significant callus formation, mimicking a tumor (Figure 13(a)). MRI examination may demonstrate nonspecific bone marrow edema (Figure 13(b)) although a search for subtle linear low signal fracture line suggests the diagnosis. Other helpful differentiating features include characteristic locations (sacrum, pubic rami, and periacetabular region) and fracture orientation (sagittally oriented sacral fractures), as well as possible bilateral symmetric findings. A CT scan may demonstrate the fracture line.

### 5.4. Particle Disease

**Figure 14:**
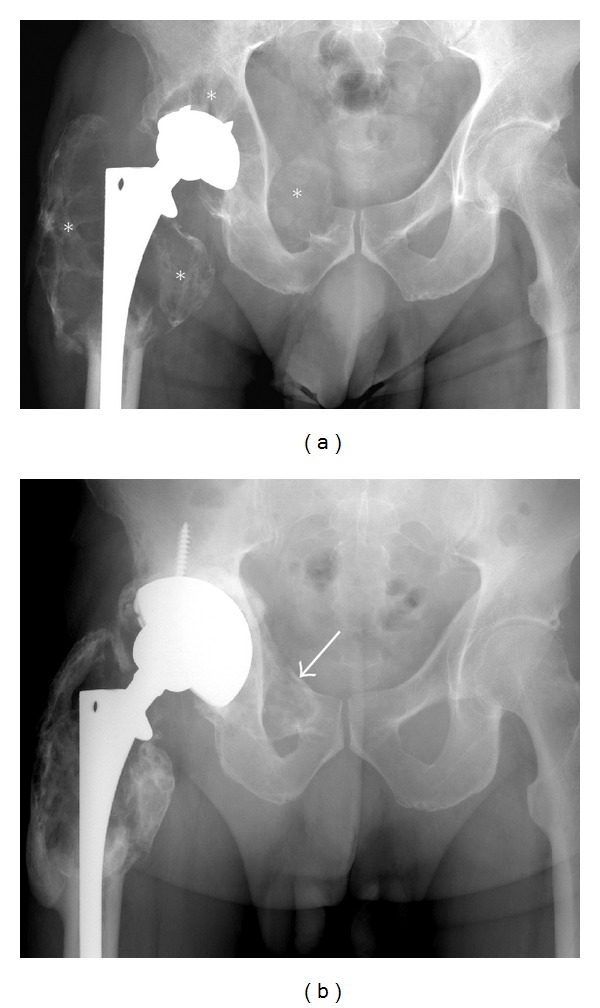
Particle disease. A frontal radiograph (a) illustrating large expansile lytic lesions (asterisk) mimicking tumors, diagnosed as particle disease. Note the proximity of these lesions to the prosthesis. The acetabular prosthesis was revised, and curettage and grafting using cancellous allograft (arrows) of the lytic areas was performed (b).

Activity-related wear and tear of a joint prosthesis sheds particles (mostly from the polyethylene liner within the acetabular cup), which induces bone resorption by activating macrophages. Among the joint replacements, it is more often seen with hip arthroplasties, ultimately leading to aseptic loosening of the prosthesis requiring revision surgery. Particle disease can mimic a lytic neoplasm (Figure 14). Common presentation is that of a lytic and sometimes expansile osseous abnormality located in close proximity to the prosthesis, occasionally associated with a soft tissue mass.

**Figure 15:**
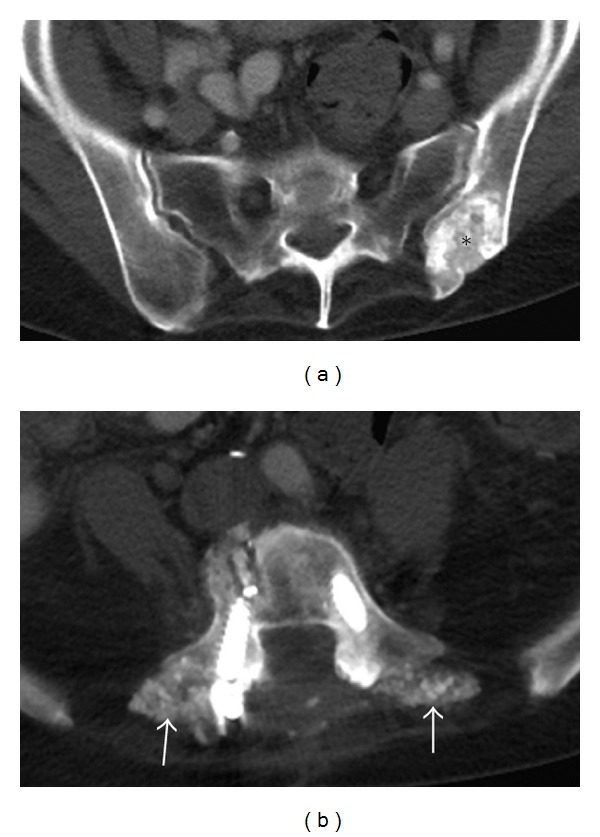
Bone graft donor site. Axial CT (a) demonstrates an aggressive appearing lesion in the left ilium, in a classic location of bone graft donor site. In this case bone graft material (arrows) was used in posterior spinal fusion surgery as shown in the axial CT (b).

**Figure 16:**
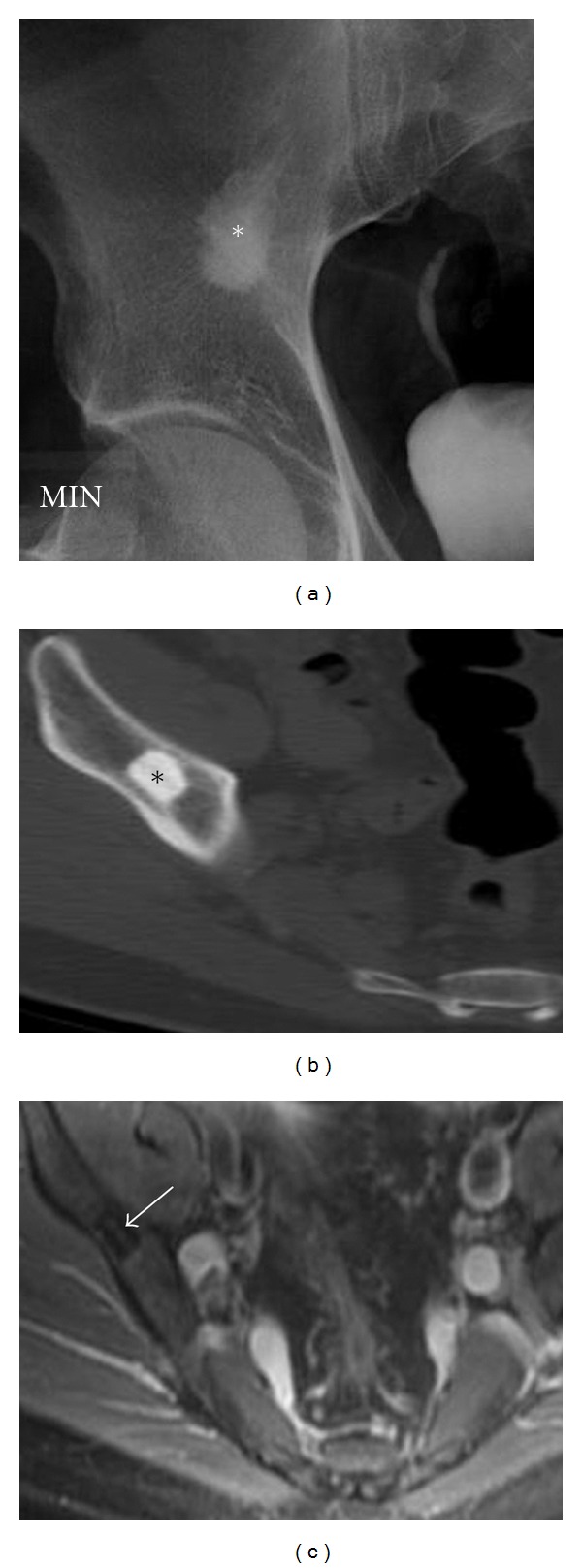
Bone island. Incidental right ilium lesion noted in a 47-year-old patient presenting with hematuria diagnosed as a bone island. AP radiograph (a) depicts a large sclerotic lesion with speculated margins blending with surrounding trabecula, a characteristic finding of a bone island (enostosis). A bone island is cortical bone in the medullary cavity (b) and hence is of low signal in all MR sequences including axial T2 MRI (c).

*A bone graft donor site* can be confused with an aggressive lesion (Figure 15) if relevant history is not available. The anatomic location of this abnormality in the posterior iliac crest is very characteristic, and looking for evidence of back surgery on scout images helps with the diagnosis. A *bone island* represents cortical bone in the medullary cavity [40] and can be of significant size in the pelvis and mimic an aggressive osseous lesion, possibly a sclerotic met. A bone island can also slowly grow in size. The presence of spiculated margins (Figures 16(a) and 16(b)), no activity or low activity on bone scan, low signal on T2-weighted images (Figure 16(c)), and lack of focal symptoms confirm the diagnosis.

## 6. Summary

In this paper, we discuss and illustrate common malignant primary and metastatic tumors that affect the skeletal pelvis, along with certain conditions that may mimic neoplastic processes. Although the differential diagnosis is extensive, appropriate use of plain radiography, CT, and MR imaging can help define anatomic extent and, along with guidance from clinical history and biopsy, aid in making the final diagnosis.
